# Associations of telemedicine vs. in-person ambulatory visits and cancellation rates and 30-day follow-up hospitalizations and emergency department visits

**DOI:** 10.1016/j.pmedr.2021.101629

**Published:** 2021-11-05

**Authors:** Julianne N. Kubes, Ilana Graetz, Zanthia Wiley, Nicole Franks, Ambar Kulshreshtha

**Affiliations:** a Office of Quality and Risk, Emory Healthcare, 478 W Peachtree St NW, Atlanta, GA, USA; b Department of Health Policy and Management, Rollins School of Public Health, Emory University, 1518 Clifton Rd, Atlanta, GA, USA; c Division of Infectious Diseases, Emory University School of Medicine, Emory University Hospital Midtown, Medical Office Tower 7th Floor, Atlanta, GA, USA; d Department of Emergency Medicine, Emory University School of Medicine, 201 Dowman Dr, Atlanta, GA, USA; e Division of Family and Preventative Medicine, Emory University School of Medicine, 201 Dowman Dr, Atlanta, GA, USA; f Department of Epidemiology, Rollins School of Public Health, Emory University, 1518 Clifton Rd, Atlanta, GA, USA

**Keywords:** Telemedicine, Telehealth, Cancellations, Patient safety, Quality improvement

## Abstract

Little is known about cancellation frequencies in telemedicine vs. in-person appointments and its impact on clinical outcomes. Our objective was to examine differences between in-person and video telemedicine appointments in terms of cancellation rates by age, race, ethnicity, gender, and insurance, and compare 30-day inpatient hospitalizations rates and 30-day emergency department visit rates between the two visit types. Demographic characteristics and comorbidities for adults scheduled for an Emory Healthcare ambulatory clinic appointment from June 2020 to December 2020 were extracted from the electronic medical record. Each appointment was identified as either a video telemedicine or in-person clinic appointment. The outcomes were ambulatory clinic cancellation rates, 30-day hospitalization rates, and 30-day emergency department visit rates. Multivariable logistic regression was used to assess differences between appointment types. A total of 1,652,623 ambulatory clinic appointments were scheduled. Ambulatory appointment cancellations rates were significantly lower among telemedicine compared to in-person appointments overall (20.4% vs. 31.0%, p < .001) and regardless of gender, age, race, ethnicity, insurance, or specialty (p < .05 for all sub-groups). Telemedicine appointments were associated with lower 30-day hospitalization rates compared to in-person appointments (AOR: 0.72, 95% CI: 0.71–0.74). There was no difference in 30-day emergency department visit rates between telemedicine and in-person appointment patients (AOR: 1.00, 95% CI: 0.98–1.02). Our findings suggest that there are fewer barriers to attending an ambulatory care visit via telemedicine relative to in-person. Using video telemedicine was not associated with more frequent adverse clinical events compared with in-person visits.

## 1 Introduction

In response to the COVID-19 pandemic, many healthcare systems rapidly developed telemedicine programs to provide ongoing access for patients to receive ambulatory care from their regular providers (Patel et al., 2021). Prior to the pandemic, payors had concerns about growth of telemedicine including the increased cost, concern for utilization, quality of care with the lack of in-person assessment, and access for disadvantaged populations. However, the pandemic accelerated rapid legislative and regulatory changes to payment and privacy requirements, supporting the unprecedented expansion of telemedicine in the United States (Mehrotra et al., 2021). With growing demand for telemedicine, major policy changes were made to support increased access, broaden coverage and widen implementation in healthcare systems. Prior studies have shown that telemedicine can safely and effectively be used to diagnose and treat acute conditions, monitor chronic conditions, conduct follow-up visits while reducing travel and wait times for patients, and potentially lower healthcare costs (Zhang et al., 2021, Dorsey and Topol, 2016, Lin et al., 2017). There is, however, limited data on telemedicine visits compared with in-person visits in regards to healthcare access and outcomes such as preventing emergency department (ED) visits and subsequent hospitalizations. Another outcome of interest is cancellation rates. Patient cancellations have a negative impact on healthcare systems leading to more costs, access issues for patients, and volatility in healthcare operations. Studying how telemedicine visits impact patient cancellation rates, ED visits, and hospitalizations is critical not only for improving health system performance and efficiency, but also for improving population health.

In the pre-COVID-19 era, there were very limited telemedicine programs in the United States with barriers such as provider reluctance, reimbursement issues, and challenges with technology use among patients. Telemedicine’s rapid adoption and expansion over the past year presents an opportunity to generate further evidence regarding its effectiveness and utility and whether it helps to improve patient outcomes (Roberts and Mehrotra, 2020). Healthcare systems and policy makers need more information on whether continuing investments in telemedicine infrastructure are worthwhile for their patients and populations. Our study is one of the first large scale studies, encompassing a diverse physician and patient population, to examine ambulatory clinic cancellations, hospitalizations, and ED visits in a major academic healthcare system. We hypothesize that compared with in-person visits, telemedicine visits have lower cancellation rates regardless of patient age, gender, race, and insurance, and similar rates of hospitalizations and ED visits.

## 2 Methods

### 2.1 Population

Emory Healthcare (EHC) is the largest healthcare system in the state of Georgia with more than 2800 physicians and 250 provider locations in urban, suburban, and rural settings. EHC has 140 group practice locations and ambulatory service sites in 27 Georgia counties, including 15 officially designated medically underserved counties. EHC began using Zoom (Zoom Video Communication, Inc., San Jose, CA) in April 2020 to offer telemedicine appointments for ambulatory care visits and included both an audio and video component in a synchronous format, which is compliant with the Health Insurance Portability and Accountability Act (HIPAA) and approved for clinical use in the United States (Compliance, 2021). . Our study included adult patients (age ≥18 years) scheduled for an ambulatory clinic appointment between June 2020 and December 2020 within EHC (when patients had a choice of in-person or telemedicine visit). We selected the above study period because appointments made between March 2020 and May 2020 were intentionally assigned to telemedicine due to the COVID-19 surge in Georgia.

### 2.2 Measures

We defined a telemedicine appointment as a visit conducted via video and an in-person appointment as a visit conducted at an ambulatory clinic when the patient was physically present. Using the Andersen framework of health services utilization, we measured predisposing factors including age, sex, gender, race, and ethnicity, enabling factors including healthcare insurance, and needs factors including present comorbidities for each patient (Andersen, 1995). All sociodemographic factors were extracted from the electronic medical record, with comorbidities identified by billed ICD-10 diagnosis codes. A Charlson Comorbidity Index (CCI) was calculated for each patient for risk adjustment (Charlson et al., 1987). We excluded patients with a positive SARS-Co-V-2 (COVID-19) polymerase chain reaction test within 14 days of their appointment, as these appointments were intentionally assigned to telemedicine to avoid risks to other patients and clinic staff. EHC patients sign a notice of privacy practices when they establish as new patients and the same practice is followed for both telemedicine and in-person clinic appointments. There are policies and systems in place to ensure patient privacy, such as ensuring telemedicine visits are conducted in a private closed room with no other individuals in the room or within hearing distance and enacting the use of waiting rooms to ensure that other individuals cannot access the visit. For data collection, no patient identifiers were used, and the study was reviewed and deemed exempt by the Emory Institutional Review Board review.

The primary process outcome was ambulatory clinic cancellation rates, defined as the percentage of ambulatory clinic appointments where the patient cancelled beforehand or did not show to the appointment. The primary clinical outcomes were 30-day hospitalization and ED visit rates, defined as the percentage of ambulatory patients who were admitted as an inpatient to a hospital or had an ED visit within 30 days of their ambulatory appointment.

### 2.3 Statistical analysis

Differences in cancellation rates between telemedicine and in-person appointments and among sub-groups were compared using the Chi-square test. Multivariable logistic regression was used to compare 30-day hospitalization and ED visit rates between telemedicine and in-person appointments, adjusting for age and CCI. Statistical analyses were performed using R (version 4.0.2; Rstudio, Inc., Boston, MA). This study followed the Strengthening the Reporting of Observational Studies in Epidemiology (STROBE) reporting guideline (Ghaferi et al., 2021).

## 3 Results

A total of 1,652,623 ambulatory clinic appointments were scheduled during the study timeframe and met the inclusion criteria. Of those, 412,936 (25.0%) were telemedicine appointments and 1,239,687 (75.0%) were in-person appointments. Physicians conducted 91% of all outpatient clinic appointments, while the other 9% were conducted by an advanced practice provider under the supervision of a physician. The average age was 59 years (SD 18.4), 61.1% were female, 47.5% were White, and 35.8% were Black. Nearly half (47.1%) of patients had commercial or private insurance. The most common comorbidities were hypertension (53.6%), followed by diabetes (23.6%) and malignancy (20.1%). Most patients (63.0%) had a low-risk CCI, defined as a CCI <2. Additional patient characteristics by appointment group are presented in Supplemental Table 1.

Ambulatory appointment cancellation rates were significantly lower among telemedicine appointments compared to in-person appointments (84,211 (20.4%) vs. 383,902 (31.0%), p < .0001, Table 1). Cancellation rates were lower for telemedicine regardless of gender, age, race, ethnicity, insurance, or specialty (p < .05 for all sub-groups).

**Table 1:** Telemedicine and In-Person Outpatient Cancellation Rates, June – December 2020.

Sub-Group	Telemedicine Appointments(n = 412,936)	In-Person Appointments (n = 1,239,687)	*P* value
Total cancellations, No. (%)	84,211 (20.4)	383,902 (31.0)	<0.0001

*Gender, No. (%)*			
Male	31,232 (20.3)	145,613 (29.8)	<0.0001
Female	25,979 (20.5)	238,267 (31.8)	<0.0001

*Age, No. (%)*			
<18	2251 (19.2)	9015 (30.2)	<0.0001
18–34	11,157 (19.6)	44,546 (30.1)	<0.0001
35–64	41,651 (20.1)	100,012 (31.3)	<0.0001
65+	29,152 (21.3)	150,329 (30.9)	<0.0001

*Race, No. (%)*			
White	29,383 (19.7)	181,464 (31.0)	<0.0001
Black	30,133 (20.5)	135,505 (30.5)	<0.0001
Asian	2571 (22.2)	118,56 (30.6)	<0.0001
American Indian/Alaska Native	199 (21.2)	1007 (30.4)	<0.0001
Native Hawaiian/Pacific Islander	167 (21.5)	853 (29.3)	<0.0001
Multiple	493 (20.3)	2110 (31.8)	<0.0001

*Ethnic Group, No. (%)*			
Non-Hispanic	63,916 (20.1)	292,156 (30.8)	<0.0001
Hispanic	2442 (22.5)	10,204 (29.7)	<0.0001

*Insurance, No. (%)*			
Commercial	38,521 (17.8)	152,115 (27.1)	<0.0001
Medicare	28,978 (19.6)	140,695 (28.9)	<0.0001
Medicaid	4002 (18.5)	16,656 (28.6)	<0.0001
Uninsured	3414 (24.5)	17,419 (25.3)	0.04

*Specialty, No. (%)*			
Primary Care	23,773 (18.1)	115,192 (32.0)	<0.0001
Sub-Specialty	54,326 (21.4)	237,748 (30.3)	<0.0001
Surgical	6112 (22.2)	30,962 (32.7)	<0.0001

Telemedicine visits were associated with lower 30-day hospitalization rate compared to in-person appointments (2.1% vs. 2.8%; OR: 0.73, 95% CI: 0.71 to 0.74, Table 2); this result did not change after adjusting for age and comorbid conditions (AOR: 0.72, 95% CI: 0.71 to 0.74). We did not find a statistically significant difference in 30-day ED visit rate between telemedicine and in-person appointments (2.6% vs. 2.6%: OR: 0.99, 95% CI: 0.96 to 1.01) after adjusting for age and comorbid conditions (AOR: 1.00, 95% CI: 0.98 to 1.02).

**Table 2:** Crude and Risk-Adjusted Clinical Outcomes for Telemedicine vs. In-Person Outpatient Appointments, June–December 2020.

Clinical Outcome	Telemedicine Appointments (n = 412,936)	In-Person Appointments Ref, (n = 1,239,687)	Odds Ratio (95% CI)	Adjusted Odds Ratio (95% CI)^a^
30-Day Hospitalizations, No. (%)	8534 (2.1)	34,984 (2.8)	0.73 (0.71, 0.74)	0.72 (0.71, 0.74)
30-Day ED Visits, No. (%)	10,543 (2.6)	32,095 (2.6)	0.99 (0.96, 1.01)	1.00 (0.98, 1.02)

^a^Adjusted for age and Charlson Comorbidity Index (CCI), which is a composite score used to predict one-year mortality. Components of CCI include myocardial infarction, congestive heart failure, peripheral vascular disease, cerebrovascular disease, dementia, chronic obstructive pulmonary disease, rheumatic disease, peptic ulcer disease, liver disease, diabetes, hemiplegia/paraplegia, renal disease, malignancy, HIV/AIDS.

## 4 Discussion

In this retrospective cohort study of adult patients receiving ambulatory care at a large academic healthcare system, telemedicine visits were associated with fewer cancellations than in-person visits during the COVID-19 pandemic; this was true for all population sub-groups. Moreover, using telemedicine was not associated with worse adverse clinical events, such as a follow-up ED visit or hospitalization.

Video telemedicine visits offer patients real-time and direct access to a clinician without leaving their homes. However, some people may face challenges with internet stability or bandwidth issues depending on internet plans and geographic location. Patients may encounter further barriers due to low socioeconomic status, infrastructure limitations, and difficulty with access due to age, disabilities, and chronic conditions that affect hearing and vision (Goins et al., 2001). A prior study found that 26.3% of Medicare beneficiaries lacked digital access at home; this was higher among those with low socioeconomic status, 85 years or older, and in communities of color (Roberts and Mehrotra, 2020). The COVID-19 pandemic has dramatically shifted usage, and telemedicine visits have been utilized more by women, older patients, and those with Medicare and Medicaid (Pierce and Stevermer, 2020). With concerns of safety in the pandemic, studies are showing that older people are more likely to adapt to telemedicine and that using telemedicine can improve access and reduce disparities (Roghani and Panahi, 2020). A recent survey study showed that utilization of and satisfaction with telehealth services was associated with regular internet access and higher health literacy regardless of age and race (Thomson et al., 2021). Several prior studies have shown that telemedicine programs hold tremendous potential for addressing rural health disparities and should continue to be prioritized (Hirko et al., 2020).

Prior concerns with telemedicine, including barriers to technology use among older and minority populations, did not seem to hold true in our study (Eberly et al., 2020). Our findings suggest that telemedicine may provide more convenient access to care and fewer barriers than in-person visits for most population sub-groups. With some training and experience, clinicians and patients may overcome challenges with technology, build rapport, and communicate appropriately in a telemedicine clinic visit, resulting in a favorable healthcare experience.

Previous studies have found that travel and wait times in clinics continue to be a significant barrier to in-person care (Ray et al., 2015). Our findings suggest that these barriers may contribute to higher cancellation rates for in-person appointments. These barriers have also been found to be more common among minority groups, resulting in disparities in healthcare access (Shi et al., 2014). Convenience of telemedicine improves access to care, particularly among vulnerable patients (Reed et al., 2020). Our results add to the literature by showing that telemedicine appointments have a higher completion rate and are not associated with higher adverse clinical events within 30 days. With fewer cancellation rates, telemedicine has the potential to improve timely appointments for prospective patients, increase operational efficiency, and reduce costs for the healthcare system.

There are several strengths to our findings. Our study was conducted at a single large healthcare system that used a common platform for most telemedicine visits during the COVID-19 pandemic. The large sample size of over 1.5 million clinic appointments provides a smaller margin of error and more closely approximates our patient population. Additionally, our sample represented a diverse patient population including age, gender, race, and geographic area.

### 4.1 Limitations

There are several limitations to the interpretation and generalizability of our findings. Our study is observational, and the results should not be interpreted as causal. Despite robust adjustment of patient characteristics, there is likely to be unmeasured confounding and our inability to know the reasons for patient cancellations. Finally, we relied on administrative and billing codes to capture visit information and there is potential for misclassification. However, it is likely to be non-differential and only bias the study results towards the null.

## 5 Conclusions

In conclusion, in a large academic health system during the COVID-19 pandemic, telemedicine appointments were cancelled significantly less than in-person appointments, regardless of age, race, ethnicity, gender, or insurance. Telemedicine appointments were associated with fewer 30-day hospitalizations compared to in-person appointments and had similar rates of ED visits. Telemedicine appointments increase access to healthcare for many sub-groups and may help reduce healthcare disparities. Expansion of telemedicine in the United States and globally warrants more efforts that focus on outcome comparisons to inform policy and clinical practice decisions. Future studies should examine differences in quality of care, patient clinical outcomes, and costs comparing telemedicine to in-person ambulatory visits more generally and for specific chronic conditions. This evidence is critical to ensure payment parity for telemedicine as compared to in-person visits for continued expansion of services for transforming healthcare delivery to improve population health.

### CRediT authorship contribution statement

**Julianne N. Kubes:** Conceptualization, Methodology, Software, Formal analysis, Investigation, Data curation, Validation, Writing – original draft, Visualization. **Ilana Graetz:** Conceptualization, Methodology, Validation, Writing – original draft, Writing – review & editing, Supervision. **Zanthia Wiley:** Methodology, Validation, Writing – original draft, Writing – review & editing. **Nicole Franks:** Resources, Writing – review & editing, Project administration, Funding acquisition. **Ambar Kulshreshtha:** Conceptualization, Resources, Writing – review & editing, Project administration, Funding acquisition.

## Declaration of Competing Interest

The authors declare that they have no known competing financial interests or personal relationships that could have appeared to influence the work reported in this paper.
