# Supplementary material for: Associations of telemedicine vs. in-person ambulatory visits and cancellation rates and 30-day follow-up hospitalizations and emergency department visits
Source: Prev Med Rep. 2021 Nov 5;24:101629. doi: 10.1016/j.pmedr.2021.101629 (PMC8684024; doi:10.1016/j.pmedr.2021.101629)
Supplement: Supplementary data 1 [file mmc1.docx]

**Supplemental Table 1.** Patient Characteristics of Telemedicine and In-Person Outpatient Appointments, June – December 2020

|  | **Telemedicine Appointments**  **(n = 412,936)** | **In-Person Appointments**  **(n = 1,239,687)** |
| --- | --- | --- |
| Gender, No. (%) |  |  |
| Male | 154,175 (37.3) | 489,245 (39.5) |
| Female | 258,761 (62.7) | 750,419 (60.5) |
| Unknown | 0 (0.0) | 26 (0.0) |
| Age, No. (%) |  |  |
| < 18 | 11,722 (2.8) | 29,835 (2.4) |
| 18-34 | 59,946 (13.8) | 147,961 (11.9) |
| 35-64 | 207,552 (50.3) | 575,966 (46.5) |
| 65+ | 136.716 (33.1) | 485,928 (39.2) |
| Race, No. (%) |  |  |
| White | 199,653 (48.3) | 585,060 (47.2) |
| Black | 147,058 (35.6) | 444,218 (35.8) |
| Asian | 11,604 (2.8) | 38,764 (3.1) |
| American Indian/Alaska Native | 940 (0.2) | 3,315 (0.3) |
| Native Hawaiian/Pacific Islander | 776 (0.2) | 2,911 (0.2) |
| Multiple | 2,425 (0.6) | 6,628 (0.5) |
| Unknown | 50,480 (12.2) | 158,794 (12.8) |
| Ethnic Group, No. (%) |  |  |
| Non-Hispanic | 318,074 (77.0) | 949,663 (76.6) |
| Hispanic | 10,862 (2.6) | 34,309 (2.8) |
| Unknown | 84,000 (20.3) | 255,718 (20.6) |
| Insurance, No. (%) |  |  |
| Commercial | 216,626 (52.5) | 561,236 (45.3) |
| Medicare | 147,864 (35.8) | 486,933 (39.3) |
| Medicaid | 24,674 (5.2) | 58,233 (4.7) |
| Uninsured | 13,941 (3.4) | 68,795 (5.5) |
| Comorbidities, No. (%) |  |  |
| Hypertension | 214,669 (52.0) | 671,601 (54.2) |
| AMI | 18,020 (4.4) | 37,055 (5.4) |
| Congestive heart failure | 46,692 (11.3) | 171,228 (13.8) |
| Peripheral vascular disease | 51,892 (12.6) | 195,501 (15.8) |
| Cerebrovascular disease | 50,432 (12.2) | 155,014 (12.5) |
| Dementia | 13,385 (3.2) | 26,823 (2.2) |
| COPD | 82,217 (19.9) | 239,311 (19.3) |
| Rheumatoid arthritis | 23,410 (5.7) | 52,755 (4.3) |
| Peptic ulcer disease | 10,406 (2.5) | 29,735 (2.4) |
| Liver disease | 52,857 (12.8) | 142,383 (11.5) |
| Diabetes | 94,199 (22.8) | 295,017 (23.8) |
| Hemiplegia/paraplegia | 9,948 (2.4) | 22,167 (1.8) |
| Renal disease | 59,640 (14.4) | 186,501 (15.0) |
| Malignancy | 70,149 (17.0) | 262,362 (21.2) |
| HIV/AIDS | 4,818 (1.2) | 15,077 (1.2) |
| Charlson Comorbidity Index, No. (%) |  |  |
| 0-1 (low risk) | 266,945 (64.6) | 774,433 (62.5) |
| 2-3 (medium risk) | 93,389 (22.6) | 294,212 (23.7) |
| 4+ (high risk) | 52,602 (12.7) | 171,045 (13.8) |
| Specialty, No. (%) |  |  |
| Primary Care | 131,353 (31.8) | 359,537 (29.0) |
| Sub-Specialty | 254,058 (61.5) | 785,511 (63.4) |
| Surgical | 27,525 (6.7) | 94,642 (7.6) |

Abbreviations: AMI, acute myocardial infarction; COPD, chronic obstructive

pulmonary disease
